# The association between cerebral “dirty-appearing” white matter and progression of small vessel disease in community-dwelling older adults

**DOI:** 10.1177/0271678X251385591

**Published:** 2025-10-22

**Authors:** Ingmar Eiling, Sigurdur Sigurdsson, Laura Verweg, Jasmin A Kuhn-Keller, Lenore J Launer, Matthias J P van Osch, Vilmundur Gudnason, Jeroen de Bresser

**Affiliations:** 1 Department of Radiology, Leiden University Medical Center, Leiden, The Netherlands; 2 Icelandic Heart Association, Kopavogur, Iceland; 3 Laboratory of Epidemiology and Population Science, National Institute on Aging, Bethesda, MD, USA; 4 Faculty of Medicine, University of Iceland, Reykjavik, Iceland

**Keywords:** Aging, cerebral small vessel diseases, magnetic resonance imaging, neuroradiology, vascular cognitive impairment

## Abstract

Cerebral “dirty-appearing” white matter (DAWM) may represent early white matter alterations linked to cerebral small vessel disease (cSVD), though its role in cSVD progression is unclear. Therefore, DAWM prevalence and associations with cSVD progression were assessed on 1.5 T brain MRI of community-dwelling older adults from the AGES–Reykjavik cohort (*n* = 2506). On baseline MRI the presence of DAWM was visually rated. DAWM prevalence was relatively high in occipital (77%) and parietal (41%) white matter and relatively low in frontal (3%) and temporal (1%) white matter. In older adults with limited baseline white matter hyperintensity (WMH) burden (*n* = 1253, selected via median split), baseline DAWM ratings were associated with an increase in total WMH volumes (*p* = 0.004) and in the parietal (*p* = 0.004) and frontal (*p* < 0.001) white matter, as well as with new subcortical infarcts (*p* = 0.018) at 5-year follow-up. DAWM was not associated with new microbleeds (*p* = 0.823) or new enlarged perivascular spaces (*p* = 0.685) at follow-up. These findings show that DAWM is prevalent in community-dwelling older adults and is associated with progression of cSVD in individuals with limited WMH burden. DAWM may therefore be an early marker of cSVD progression and may help to identify older adults for personalized lifestyle interventions to prevent cognitive decline.

## Introduction

Cerebral small vessel disease (cSVD) in older adults is a major contributor to long-term cognitive decline^1^ and dementia,^2^ and often co-occurs with Alzheimer’s disease.^3^ Being able to estimate the progression of cSVD early on, using MRI biomarkers, is therefore important to determine prognosis. Furthermore, this could be helpful in early identification of older individuals at increased risk of cSVD progression for personalized lifestyle interventions and may aid in participant selection and disease monitoring for clinical trials.

Conventional neuroimaging markers of cSVD include white matter hyperintensities (WMH) of presumed vascular origin, lacunes, microbleeds, and enlarged perivascular spaces.^4,5^ However, these often represent late changes of cSVD and are therefore less suited as early markers that could predict disease progression. In another disease (multiple sclerosis) diffuse, sub-hyperintense white matter areas on FLAIR MRI that were once considered normal-appearing white matter are now considered as “diffusely abnormal” or “dirty-appearing” white matter (DAWM). Furthermore, these areas are related to multiple sclerosis disease progression and white matter lesion growth.^6^ Advancement in modern 1.5 and 3 T MRI systems have led to this increased detection rate of DAWM.^7^ DAWM consists of nonfocal T2 signal intensities higher than normal-appearing white matter, but lower than WMH.^7,8^ Importantly, in community-dwelling older adults, such white matter signal abnormalities can also be observed despite the pathophysiological changes being different than in multiple sclerosis. DAWM might therefore also be related to cSVD progression in older adults, but no previous studies have assessed this potential relationship.

Our hypothesis is that visually observed DAWM is an early marker of cSVD, and as such would precede conventional cSVD changes in older adults. The aims of our study were therefore to assess the prevalence of DAWM in a large cohort of community-dwelling older adults and investigate whether DAWM is associated with long-term progression of cSVD markers in individuals with limited baseline cSVD burden.

## Methods

### Study design and participants

Data was available from 4614 participants of the Icelandic AGES–Reykjavik prospective cohort study who were enrolled from the prior Reykjavik Study by the Icelandic Heart Association. Baseline data and MRI scans were collected on a 1.5 T MRI at a study center in Reykjavik between 2002 and 2006,^9^ with follow-up scan acquisition between 2007 and 2011.^10^ All participants signed written informed consent prior to participating in the baseline and follow-up study. The study was approved by the Institutional Review Board of Iceland (IRB, VSN 00-063), the Icelandic Data Protection Committee, and the IRB serving the National Institute on Aging, in accordance with the Declaration of Helsinki.^9^

Two thousand five hundred six participants that underwent both baseline and follow-up scans were included in the current study (Figure 1). Mean time to follow-up was 5.2 ± 0.2 (SD) years. Participants were excluded (*n* *=* 53) if their MRI scan contained high noise, artefacts such as Gibbs ringing, or if large parts of the white matter showed pathological changes such as infarcts, for example, by observing hyperintense patterns connected to the cortex. We did not exclude cases of minor white matter atrophy or minor ventriculomegaly.

**Figure 1.**
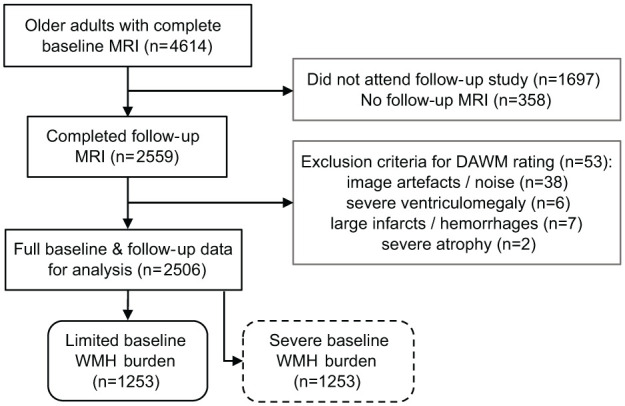
Flowchart of exclusion criteria for the study group. Separate analyses were conducted on two groups after a median split of the total study group into a limited and severe baseline WMH burden group.

### Procedures

Baseline participant information was collected using questionnaires by research staff.^9,10^ The participant’s highest education level was recorded as primary school, secondary school, college, or university. Former tobacco smokers had smoked at least 100 cigarettes or 20 cigars, non-smokers smoked less than that or never smoked, and current smokers were smoking actively at baseline. Body mass index (BMI) was calculated from self-reported height and weight. Systolic and diastolic blood pressure were averaged over two measurements with a standard mercury sphygmomanometer. Hypertension status was defined by self-report, antihypertensive medication use or by >140 mmHg systolic and/or >90 mm diastolic blood pressure. Diabetes mellitus status was defined by self-report, antidiabetic medication use or fasting blood glucose >7 mmol/L. Coronary artery disease was defined by self-report, angina plus nitrate use or electrocardiogram evidence of myocardial infarction.

### MRI acquisition protocol

MR images were acquired on a 1.5 T Signa Twinspeed EXCITE (GE, Waukesha, WI, USA) with a four- or eight-channel phased array head coil. Standardized protocol sequences included a 3D T1-weighted spoiled gradient echo (TE/TR = 8/21 ms, FA 30°, 240 mm field of view, 256 × 256 matrix, 0.94 × 0.94 × 1.5 mm voxels), T2-weighted fast spin-echo fluid attenuated inversion recovery (FLAIR; TE/TR/TI = 100/8000/2000 ms), T2*-weighted gradient echo echo-planar imaging (TE/TR = 50/3050 ms), and a proton density/T2-weighted fast spin-echo (TE1/TE2/TR = 22/90/3220 ms, ETL = 8). The latter three scans were acquired with a 90° flip angle, 256 × 256 matrix, 220 mm field of view, and 0.86 × 0.86 × 3.0 mm voxels with interleaved slices, angulated parallel to the anterior-posterior commissure. The same scanner and protocols were used for baseline and longitudinal follow-up MRI measurements.

### Dirty-appearing white matter rating

Before rating, T1-weighed images (T1WI) were registered (affine) to 2009c MNI space without reslicing, using ITK-Elastix^11^ 0.10 in Python 3.8.5. FLAIR images were co-registered and resliced to the normalized T1WI for more consistent rating. No registration distortions were detected in the registered FLAIR images.

We chose to visually estimate DAWM since its diffuse, low-contrast appearance makes volumetric segmentation challenging and time-consuming and automated segmentation algorithms for DAWM are not currently available for this population. DAWM was visually rated relative to the apparent volume of lobar white matter,^8^ defined via landmarks. We did so separately for the bilateral frontal, temporal, parietal, and occipital lobes using a visual rating scale (0: no DAWM, 1: >0%–10% of lobar white matter, 2: >10%–25% of lobar white matter, 3: >25%–50% of lobar white matter, 4: >50%–75% of lobar white matter, 5: >75% of lobar white matter), and also added these ratings into a sum rating (range 0–20).

DAWM was defined as an area of diffuse, subtle FLAIR signal hyperintensity with a minimal axial diameter of 3 mm, visible on at least two axial slices, and with a 3 mm distance from WMH edges in the axial and sagittal plane to avoid confusing DAWM with partial voluming of WMH edges (see Figures 2 and 3). DAWM was distinguished from isointense white matter structures (e.g. corona radiata, thalamocortical projections, and optic radiations fanning out near the cortex) by identifying an irregular shaped yet soft edge. DAWM was not rated when connected to the cortex, indicating cortical infarcts. Criteria were defined in collaboration with an experienced neuroradiologist (JdB) and consensus meetings were regularly performed. DAWM was rarely rated in the presence of large confluent WMH, which are most common in frontal and parietal white matter areas (i.e. centrum semiovale). This was due to our avoidance of partial voluming from WMH edges and because WMH could take up a large part of the white matter in which DAWM could occur. In contrast, parietal DAWM was frequently observed near multiple focal deep WMH. Frontal DAWM was observed less often, as periventricular WMH caps often sharply transitioned into normal-appearing white matter with no sign of DAWM as intermediate stage.

**Figure 2.**
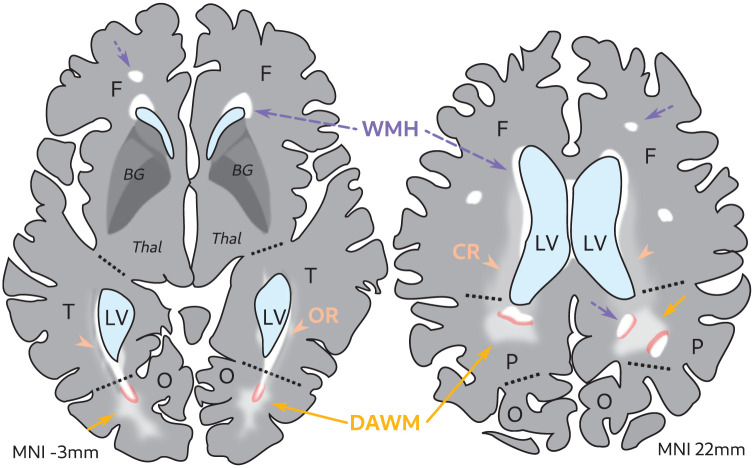
Schematic of DAWM rating in the presence of WMH. Illustration of a FLAIR MRI scan of an older adult brain. DAWM (yellow solid arrows) can be attached to WMH (white areas, purple arrows dotted lines) without a clear border, hence the transition zones (in pink, 3 mm) that were disregarded during rating. Note that some FLAIR signal changes can be attributed to white matter tract bundles such as the CR (orange arrowheads) and OR (orange arrowheads). BG: basal ganglia; F: frontal; LV: lateral ventricles; MNI: Montreal Neurological Institute template space; O: occipital; P: parietal; T: temporal; Thal: thalamus; CR: corona radiata; OR: optic radiation.

**Figure 3.**
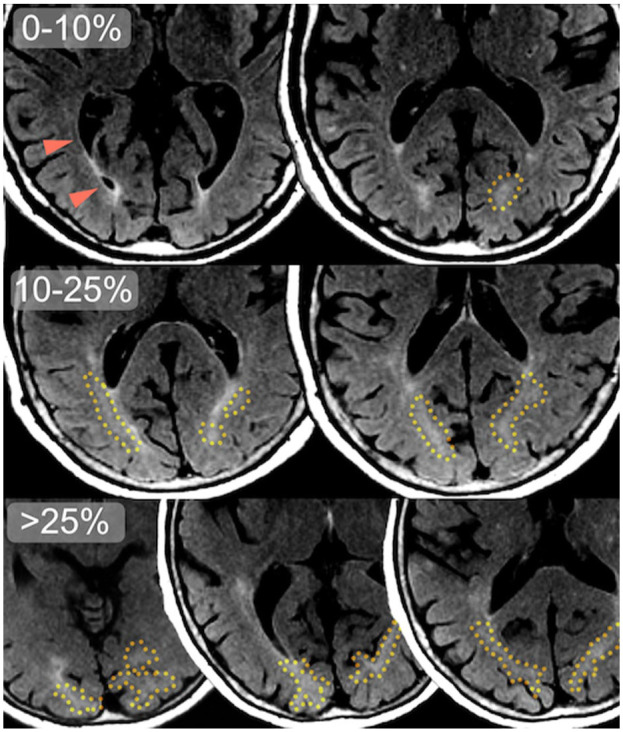
Common locations of occipital DAWM on FLAIR MRI slices. Transversal slices of 1.5 T FLAIR MRI scans of three different older adult participants, with DAWM denoted by a yellow dotted line. Note the difference in apparent DAWM volume between the top row (0%–10% DAWM of total occipital white matter), where only small occipital periventricular WMH and ependymal lining is visible, and the middle row (10%–25% DAWM) and bottom row (>25% DAWM) where larger, bilateral more confluent configurations of occipital DAWM can be seen, extending beyond the slices shown here. The optic radiation is denoted by orange arrowheads: its darker anterior segment is Meyer’s loop curving around the ventricles, while its fanning connections to the cortex taper off with a very subtle, diffuse intensity gradient.

Two raters, IE and LV, performed the DAWM rating in an in-house tool built in MATLAB 2020a (MathWorks, Natick, MA, USA) with fine-tunable window-level adjustments and automated rating logging. Raters were blinded to any patient characteristics. Intra-rater reliability for the lobar DAWM ratings was good for IE (linear weighted κ = 0.72 over several months, 95% CI (0.60–0.85), *n* *=* 70), who rated 1341 scans and assessed all scan exclusions, and excellent for LV (κ = 0.91 over 2 weeks, 95% CI (0.83–0.98), *n* *=* 24) who rated 1165 scans. The inter-rater reliability between IE and LV was good across all lobar ratings (κ = 0.72, 95% CI (0.63–0.82), *n* *=* 100).

### cSVD MRI markers

Gray matter, white matter, cerebrospinal fluid, and WMH volume were segmented for both baseline and follow-up scans using a modified algorithm based on the MNI pipeline^12^ and later parcellated per white matter region.^13^ Total intracranial volume was defined as the sum of these volumes. Cerebral microbleeds, infarcts, and enlarged perivascular spaces (ePVS) were counted by two trained radiographers.^10,14^ ePVS were classified as round or tubular defects larger than 3 mm on its shortest axis with no adjacent hyperintensities or hemosiderin evidence.^15^ These markers were counted as new if they were only present on the follow-up scan but not on the baseline scan.

### Statistical analysis

Lobar DAWM ratings (*n* = 2506) were used to determine its prevalence. DAWM sum ratings were associated with age using linear regression, and the difference in DAWM between males and females was tested with an independent *t-*test.

As we were interested in DAWM as an early marker of cSVD, we assessed DAWM in a limited cSVD burden group by performing a median split on baseline WMH volume (normalized by total intracranial volume) to divide the cohort into two groups with limited and severe baseline WMH burden. Between-group differences in participant characteristics were calculated via independent *t*-tests or χ^2^ tests.

In our primary analysis of the limited baseline WMH burden group (*n* *=* 1253), we tested the association between DAWM at baseline with WMH volume increase at follow-up using linear regression analyses. The association between DAWM at baseline and any new subcortical infarcts, new microbleeds, and new ePVS at follow-up was tested using logistic regression analyses. These marker counts are highly skewed—most participants have low progression counts, whereas very few have high counts—and were therefore binarized to suit a logistic regression approach. Statistical models were adjusted for age, sex, total intracranial volume (for WMH volume changes), and vascular risk factors (hypertension, type 2 diabetes mellitus, smoking status, and body mass index). As secondary analyses, adjusted linear regression models were also performed per lobe. Lobar WMH volume changes at follow-up were calculated by subtracting the baseline and follow-up WMH volumes gained from atlas parcellation.^13^

To provide a frame of reference for our DAWM analyses, we also studied the association of baseline WMH volume with WMH volume increase (linear regression analyses adjusting for age, sex, total intracranial volume, and vascular risk factors) as well as with any amount of new subcortical infarcts, new microbleeds, and new ePVS at follow-up (logistic regression analyses adjusting for age, sex, and vascular risk factors). Furthermore, sensitivity analyses were conducted to test whether baseline DAWM ratings explain variance above baseline WMH volume (Supplemental Materials).

As secondary analyses, we tested the association of DAWM at baseline with WMH volume increase as well as new microbleeds, ePVS, and subcortical infarcts at follow-up in the severe baseline WMH burden group (*n* = 1253) in the same way as for our primary analyses.

Data preparation and analysis was performed in RStudio with R version 4.3.3.

## Results

### Prevalence of DAWM in community-dwelling older adults

Participants with follow-up brain MRI were on average 74.6 ± 4.8 (SD) years old at baseline and 59% were female. 2050 of 2506 participants (82%) showed any DAWM on brain MRI of which 849 (34%) with a DAWM sum rating of 1, 708 (28%) with a sum rating of 2, 339 (14%) with a sum rating of 3, 110 (4%) with a sum rating of 4, 34 (1%) with a sum rating of 5, 8 (0.003%) with a sum rating of 6, and 2 (0.0008%) with a sum rating of 7 (see Figure 4(a)). 456 participants (18%) had no observable DAWM. The mean DAWM sum rating was 1.58 ± 1.18 (SD). DAWM was most prevalent in the occipital white matter (*n* *=* 1940, 77%), less prevalent in the parietal white matter (*n* *=* 1035, 41%), and uncommon in the frontal white matter (*n* *=* 79, 3%) and temporal white matter (*n* *=* 25, 1%). Baseline DAWM sum rating did not show a significant association with baseline age (*B*: −0.06 (−0.10 to 0.22), *p* = 0.430) or sex (*p* = 0.196).

**Figure 4.**
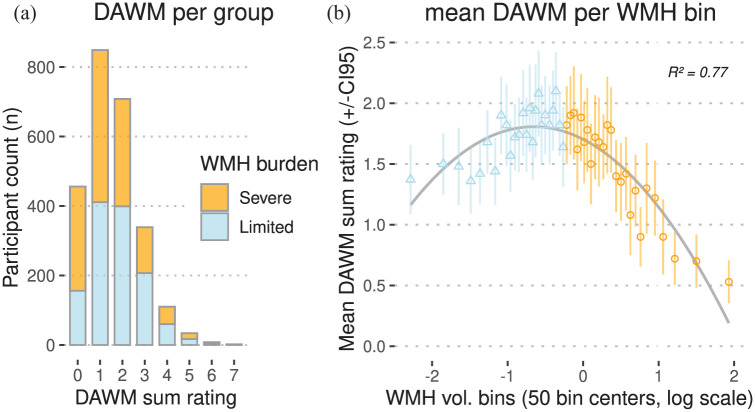
Distributions of DAWM in community-dwelling older adults (*n* *=* 2506). (a) Stacked bar chart of DAWM sum ratings on the *x*-axis and participant counts on the *y*-axis, divided into severe and limited baseline WMH burden groups (*n* *=* 1253 each). (b) Bins of baseline WMH volume normalized by total intracranial volume on the *x*-axis, versus mean DAWM sum rating per bin on the *y*-axis. Each of the 50 bins (containing *n* *=* 50 or *n* *=* 51 individuals) represents 2% of the distribution. Bins are centered on a natural log scale: leftmost bins contain near-zero WMH volumes, rightmost bins contain large WMH volumes. *R*^2^ shows a good least-squares quadratic fit of the bin points, shown by the inverse U-shaped line in gray. CI95: 95% confidence intervals of bin averages.

Binning the baseline DAWM sum ratings showed a visual inverse U-shaped (quadratic) relation between mean DAWM per bin and baseline WMH burden (Figure 4(b)). This was likely caused by the reduced normal-appearing white matter volume in which DAWM could manifest in individuals with severe WMH burden. This was especially apparent surrounding the lateral ventricles as this is a common location for both DAWM and WMH (Figures 2 and 3). As we were primarily interested in DAWM as an early marker of cSVD progression, a median split (at 0.79% WMH of total intracranial volume) was performed to select a group with limited baseline WMH burden (Figure 4). The mean DAWM sum rating was significantly higher in the limited baseline WMH burden group compared to the severe baseline WMH burden group (mean difference: 0.31 (0.22–0.40), *T*: 6.69, *p* < 0.001; Table 1). The limited baseline WMH burden group is significantly younger and has a lower prevalence of vascular risk factors (hypertension, coronary artery disease, type 2 diabetes mellitus, and tobacco use) compared to the severe baseline WMH burden group (Table 1).

**Table 1.** Baseline characteristics of the study group.

Baseline statistic	Limited baseline WMH burden (*n* *=* 1253)	Severe baseline WMH burden (*n* *=* 1253)	*p* value	Total group (*n* *=* 2506)
Age (years)	73.6 ± 4.4	75.7 ± 4.8	<0.001	74.6 ± 4.8
Female	763 (61%)	721 (58%)	0.096	1484 (59%)
Education level	2.2 ± 0.9	2.2 ± 0.9	0.768	2.2 ± 0.9
BMI (kg/m^2^)	27.2 ± 4.0	27.2 ± 4.1	0.423	27.2 ± 4.1
Hypertension	915 (73%)	1031 (82%)	<0.001	1945 (78%)
Coronary artery disease	166 (13%)	261 (21%)	<0.001	427 (17%)
Diabetes mellitus type 2	95 (8%)	128 (10%)	0.012	223 (9%)
Never used tobacco	590 (47%)	506 (40%)	<0.001	1096 (44%)
Former tobacco user	552 (44%)	586 (47%)	0.160	1138 (45%)
Current tobacco user	111 (9%)	161 (13%)	<0.001	272 (11%)
DAWM sum rating	1.7 ± 1.1	1.4 ± 1.2	<0.001	1.6 ± 1.2
WMH volume (ml)	6.6 (4.5–8.7)	21.7 (15.5–32.0)	n.a.	11.7 (6.6–21.7)

Values are given as mean ± standard deviation, *n* (%), or median (interquartile range). Education levels range between 1 and 4. Age (years), BMI (kg/m^2^), education level differences, and mean DAWM sum rating were compared between groups using two-sided *t*-tests, while χ^2^ tests were performed for the other variables. A significance test on WMH volume row per group is not applicable because the two groups were made by splitting the dataset on WMH volume.

BMI: body mass index; n.a.: not applicable.

Baseline DAWM and long-term progression of cSVD markers in the limited baseline WMH burden group

The limited baseline WMH burden group (*n* *=* 1253) had a median baseline WMH volume of 6.6 ml (IQR: 4.5–8.7). At 5.2-year follow-up this increased to 8.5 ml (IQR: 5.0–11.9; Table 2). Within this group, a higher baseline DAWM sum rating was associated with a larger increase in WMH volume at follow-up (*B* (95% CI): 0.26 ml (0.08–0.45), *p* = 0.004; Table 2). Not adjusting for vascular risk factors showed comparable results (*B*: 0.26 ml (0.08–0.44), *p* = 0.005). As a frame of reference, baseline WMH volume showed a similar association with a larger increase in WMH volume at follow-up (*B*: 0.38 ml (0.31–0.45), *p* < 0.001).

**Table 2.** Associations of baseline DAWM with global and lobar WMH volume progression in the limited baseline WMH burden group (*n* *=* 1253).

Region	Baseline (mean ± SD)	Follow-up (mean ± SD)	Volume change (mean ± SD)	Primary model (*B* ± 95% CI)	Secondary model (*B* ± 95% CI)
Global WMH (ml)	6.6 ± 2.9	9.1 ± 5.4	2.5 ± 3.8	0.26 (0.08–45)*	0.26 (0.08–0.44)*
Frontal WMH (ml)	2.3 ± 1.4	3.5 ± 2.5	1.2 ± 1.6	1.04 (0.45–1.63)*	1.04 (0.46–1.63)*
Parietal WMH (ml)	0.9 ± 0.7	1.4 ± 1.3	0.5 ± 0.9	0.12 (0.04–0.20)*	0.11 (0.03–0.19)*
Temporal WMH (ml)	0.6 ± 0.5	0.9 ± 0.9	0.3 ± 0.6	0.09 (−0.34 to 0.52)	0.09 (−0.34 to 0.52)
Occipital WMH (ml)	1.9 ± 1.1	2.5 ± 1.6	0.6 ± 0.8	0.01 (−0.05 to 0.07)	0.00 (−0.06 to 0.06)

*B* values (95% CI) indicate WMH volume changes (ml) from baseline to follow-up per unit increase in baseline DAWM rating. Mean and SD of volume change were calculated from subtracted follow-up and baseline volumes (its delta). DAWM sum ratings were associated with global WMH volumes while lobar DAWM ratings were associated with the respective lobar WMH volumes. Both models were adjusted for age, sex, and total intracranial volume, while the primary model was additionally adjusted for vascular risk factors (hypertension, type 2 diabetes mellitus, smoking status, and BMI).

* *p* < 0.05.

Sensitivity analyses were performed by selecting a subgroup with very low baseline WMH volumes (bottom quartile) and combining both DAWM and WMH in a single model (Supplemental Materials). In this very low baseline WMH volume subgroup, contrary to the limited WMH burden group, higher DAWM sum ratings were associated with larger increase in WMH volume at follow-up, suggesting that DAWM ratings explain variance above baseline WMH volumes (Supplemental Table S3).

In secondary analyses of DAWM per lobe within the limited baseline WMH burden group, higher baseline DAWM ratings were associated with a larger increase in WMH volume at follow-up in the parietal white matter (*B* (95% CI): 0.12 ml (0.04–0.20), *p* = 0.004) and the frontal white matter (*B*: 1.04 ml (0.45–1.63), *p* < 0.001). Higher DAWM ratings showed no association in the occipital white matter (*B*: 0.01 ml (−0.05 to 0.07), *p* = 0.823) or temporal white matter (*B*: 0.09 ml (−0.34 to 0.52), *p* = 0.685).

Regarding the other cSVD markers, at follow-up 22 participants (2%) had new subcortical infarcts, 160 participants (13%) had new microbleeds, and 27 participants (2%) had new ePVS (Table 3). A higher baseline DAWM sum rating showed an association with increased odds of new subcortical infarcts at follow-up (OR (95% CI): 1.50 (1.06–2.10), *p* = 0.018), but showed no association with new microbleeds (OR: 1.05 (0.99–1.12), *p* = 0.120) or new ePVS (OR: 1.06 (0.75–1.46), *p* = 0.737) at follow-up (Table 3). Not adjusting for vascular risk factors showed comparable results. As a frame of reference, a higher baseline WMH volume was also associated with increased odds of new subcortical infarcts at follow-up (OR: 1.20 (1.03–1.43), *p* = 0.025), but not with new microbleeds (OR: 1.25 (0.97–1.61), *p* = 0.089) or new ePVS (OR: 1.05 (0.91–1.21), *p* = 0.532) at follow-up.

**Table 3.** Associations of baseline DAWM with subcortical infarcts, microbleeds, and ePVS progression in the limited baseline WMH burden group (*n* *=* 1253).

cSVD marker	Baseline (*n* (%) individuals)	Any new at FU (*n* (%) individuals)	Total (*n* (%) individuals)	Primary model (OR (95% CI))	Secondary model (OR (95% CI))
Subcortical infarcts	34 (2.7%)	22 (1.7%)	50 (4.0%)	1.50 (1.06–2.10)*	1.48 (1.05–2.06)*
Microbleeds	167 (13.3%)	160 (12.8%)	273 (21.8%)	0.97 (0.83–1.12)	0.97 (0.83–1.12)
ePVS	159 (12.7%)	27 (2.2%)	166 (13.3%)	0.88 (0.60–1.24)	1.07 (0.77–1.48)

FU: follow-up.

OR (95% CI) indicate the odds ratios per unit increase in baseline DAWM sum rating. Total (*n* (%)) individuals refers to the *n* individuals that either had a marker at baseline or a new marker at *FU*, but not at both timepoints, as to not count individuals twice. The secondary model was adjusted for age and sex. The primary model was additionally adjusted for vascular risk factors (hypertension, type 2 diabetes mellitus, smoking status, and BMI).

* *p* < 0.05.

### Secondary analyses in the severe baseline WMH burden group

As expected, the severe baseline WMH burden group were observed with more subcortical infarcts, microbleeds, and ePVS at baseline compared to the limited baseline WMH burden group (see Supplemental Tables S1 and S2). For the severe baseline WMH burden group, higher baseline DAWM sum ratings showed an association with a lower increase in WMH volume at follow-up (*B* (95% CI): −1.03 ml (−1.48 to −0.59), *p* < 0.001), but did not show an association with increased odds of new subcortical infarcts (OR (95% CI): 1.03 (0.85–1.22), *p* = 0.775), new microbleeds (OR (95% CI): 0.94 (0.84–1.05), *p* = 0.286), or new ePVS (OR (95% CI): 0.96 (0.74–1.21), *p* = 0.744).

## Discussion

Our study shows that cerebral DAWM has a high prevalence in a large cohort of community-dwelling older adults, especially in the parietal and occipital lobar white matter. A higher DAWM rating at baseline in community-dwelling older adults with limited WMH burden was associated with a higher WMH volume increase at the 5.2-year follow-up as well as higher odds of new subcortical infarcts at follow-up, but was not associated with new ePVS or new microbleeds at follow-up.

Few previous studies have assessed the prevalence of DAWM in community-dwelling older adults and none have associated DAWM with cSVD progression. One previous cross-sectional study^16^ that investigated FLAIR MRI intensity profiles and their association with vascular risk factors classified 10% of community-dwelling older adults (*n* *=* 665; age range 72–73 years) as having extensive “dirty,” “diffuse,” or “subtle” periventricular hyperintensities in the centrum semiovale. However, their classification method is not directly comparable to our rating system as the presence of DAWM was only noted when WMH categories did not apply, even though both can co-exist, and DAWM was only rated in a single location. Another cross-sectional study^17^ (*n* *=* 101) quantified DAWM volume by manual segmentation of uniform nonfocal signal increases with intensities between the contralateral normal-appearing white matter and WMH. They found a mean DAWM volume of 0.9 ± 1.1 ml (SD) in neurologically healthy participants aged 18–75 (mean age 44.7 ± 17.8). Lastly, a small pilot study^18^ described DAWM and WMH in relation to jugular venous reflux in Alzheimer’s disease (*n* *=* 12), mild cognitive impairment (*n* *=* 24), and matched controls (*n* *=* 17). DAWM was defined as a nonfocal areas of subtle signal increase compared to normal appearing white matter. They found a mean of 0.9 ± 0.9 ml DAWM in the control group (mean age 81.4 ± 3.8).

The pathological correlates of DAWM in cSVD are unclear. More is known about the pathological correlates of WMH: they represent a core of white matter injury containing extracellular water^19–22^ which stems from pathology including myelin attenuation and eventual loss of axons.^20,23,24^ WMH are presumed to largely be caused by vascular dysfunction and tissue hypoxia (e.g. from arteriolosclerosis^25^). Myelin attenuation has also been found in white matter areas *absent* of definitive WMH on post-mortem T2-weighted brain MRI (showing “subtle, diffuse changes of slightly increased signal intensity”).^26^ On slices of 33 brains from community-dwelling older adults, these subtle white matter signal abnormalities contained lesser degrees of capillary endothelial and glial activation alongside myelin attenuation than within definitive WMH. As the definition of diffuse, subtle white matter abnormalities in this previous study show similarities to our definition of DAWM, it is possible that DAWM areas could be related to pathological processes similar, yet more benign, than found in WMH.

Long-term progression of WMH volume is strongly associated with occurrence of new lacunes, and new lacunes often touch on preexisting WMH.^27^ The current study also investigated the progression of new subcortical infarcts, which mostly represent lacunes. We found that DAWM (and baseline WMH volume) was related to increased odds of new subcortical infarcts at follow-up in individuals with limited baseline WMH burden. It is possible that DAWM reflects white matter pathology, that is, linked to later subcortical infarcts such as lacunes in the white matter. However, DAWM ratings did not relate to occurrence of new ePVS or new microbleeds at follow-up. One possibility is that 1.5 T MRI is only sensitive to larger ePVS and microbleeds, which might have obscured the full progression of these small abnormalities. Alternatively, perhaps there is a specific pathological process of cSVD such as hypoperfusion that underlies the development of DAWM, WMH, and subcortical infarcts, that is, less strongly associated with ePVS and microbleeds.

In our study the visual DAWM ratings showed an inversed U-shaped (quadratic) relation with baseline WMH volume (Figure 4(b)). As DAWM cannot occur in the same location as WMH, and as they are both often found in deep watershed areas near the posterior lateral ventricles, it is possible that DAWM could precede WMH formation in these areas. In line with this observation, frontal and parietal DAWM near the ventricles was related to local WMH volume increases in individuals with limited WMH burden. DAWM ratings in cSVD thus seem most useful in individuals with only limited WMH burden, with special attention to the frontal and parietal white matter.

Strengths of our study include our estimates of DAWM prevalence and its relation to long-term cSVD progression in a large and well-phenotyped community-dwelling longitudinal cohort. This gives our study significant external validity towards community-dwelling older adults. Ratings were kept unbiased by blinding raters to any participant characteristics and randomizing participant order.

Our study has some limitations. First of all, many baseline participants did not have follow-up data available.^10^ As drop-out chance increases with declining health, the follow-up was biased towards healthier individuals with less vascular risk factors^28^ which leads to underestimation of the true rate of cSVD progression in the population. However, our selection of individuals with limited baseline cSVD burden were already in better health compared to the full cohort. Hence, bias in health status at follow-up likely affects our results to a much lesser degree. Medical interventions between baseline and follow-up could also have influenced the observed rate of cSVD progression. We have adjusted our analyses for hypertension and type 2 diabetes mellitus status to try to mitigate this influence on the analyses. Furthermore, visual rating of DAWM can be challenging as it is based on gradual intensity transitions ranging in-between WMH and normal-appearing white matter in the presence of scan inhomogeneities. This resulted in a good (but not excellent) inter-rater reliability (κ = 0.72) taking into account specific mimics, though further improving agreement between raters was challenging given the previously mentioned reasons. Lastly, a limitation could be that the 1.5 T MRI scans that we used might have a more limited tissue contrast to observe DAWM in certain regions compared to higher field strength MRI scans with newer hardware. Despite this limitation, however, we were able to find statistically significant associations in our data.

In conclusion, cerebral DAWM has a high prevalence in community-dwelling older adults, especially in the occipital and parietal white matter. The association of DAWM with global and lobar progression of WMH as well as with any new subcortical infarcts indicates that DAWM could be an early marker of cSVD in individuals with limited baseline WMH burden. DAWM could therefore be helpful in early identification of individuals at increased risk of cSVD progression who may benefit from personalized lifestyle interventions.

## Supplemental Material

sj-docx-1-jcb-10.1177_0271678X251385591 – Supplemental material for The association between cerebral “dirty-appearing” white matter and progression of small vessel disease in community-dwelling older adults Supplemental material, sj-docx-1-jcb-10.1177_0271678X251385591 for The association between cerebral “dirty-appearing” white matter and progression of small vessel disease in community-dwelling older adults by Ingmar Eiling, Sigurdur Sigurdsson, Laura Verweg, Jasmin A Kuhn-Keller, Lenore J Launer, Matthias J P van Osch, Vilmundur Gudnason and Jeroen de Bresser in Journal of Cerebral Blood Flow & Metabolism
