# Supplementary material for: The association between cerebral “dirty-appearing” white matter and progression of small vessel disease in community-dwelling older adults
Source: J Cereb Blood Flow Metab. 2025 Oct 22;46(3):764–72. doi: 10.1177/0271678X251385591 (PMC12546102; doi:10.1177/0271678X251385591)
Supplement: sj-docx-1-jcb-10.1177_0271678X251385591 – Supplemental material for The association between cerebral “dirty-appearing” white matter and progression of small vessel disease in community-dwelling older adults [file sj-docx-1-jcb-10.1177_0271678X251385591.docx]

Supplements to: The association between cerebral “dirty-appearing” white matter and progression of small vessel disease in community-dwelling older adults

Ingmar Eiling, MSc^1*^, Sigurdur Sigurdsson, PhD^2^, Laura Verweg, BSc^1^, Jasmin A. Kuhn-Keller, PhD^1^, Lenore J. Launer, PhD^3^, Matthias J. P. van Osch, PhD^1^, Vilmundur Gudnason, PhD^2,4^, Jeroen de Bresser, PhD^1^

^1^Department of Radiology, Leiden University Medical Center, Leiden, The Netherlands, ^2^Icelandic Heart Association, Kopavogur, Iceland, ^3^Laboratory of Epidemiology and Population Science, National Institute on Aging, Bethesda, Maryland, USA, ^4^Faculty of Medicine, University of Iceland, Reykjavik, Iceland

* Correspondence to: Ingmar Eiling, C.J. Gorter MRI Center, Leiden University Medical Center, Albinusdreef 2, 2333 ZA Leiden, The Netherlands. Email: i.eiling@lumc.nl

**cSVD markers in the limited and severe baseline WMH burden groups**

|  |  | | |  |  |  |  |
| --- | --- | --- | --- | --- | --- | --- | --- |
|  | *Limited baseline WMH burden group (n=1253)* | | |  | *Severe baseline WMH burden group (n=1253)* | | |
|  | *Baseline vol.* | *Follow-up vol.* | *Volume change* |  | *Baseline vol.* | *Follow-up vol.* | *Volume change* |
| *WMH (ml) mean* ± SD | 6.6 ± 2.9 | 9.1 ± 5.3 | 2.5 ± 3.8 |  | 28.0 ± 20.0 | 37.7 ± 25.0 | 9.7 ± 9.7 |
| *WMH (ml) median [IQR]* | 6.6  [4.5, 8.7] | 8.5  [5.0, 11.9] | 1.6  [0.2, 4.0] |  | 21.7  [15.6, 32.0] | 29.9  [21.0, 46.0] | 8.0  [3.7, 13.2] |

**Table S1: WMH marker changes per group.** Change in WMH volumes over the follow-up period for both the limited and severe baseline WMH burden group. Mean (SD) and median [IQR[ of volume change were calculated on subtracted volumes from follow-up and baseline (its delta). WMH: white matter hyperintensities.

|  | Limited baseline WMH burden group (n=1253) | | |  | Severe baseline WMH burden group (n=1253) | | |
| --- | --- | --- | --- | --- | --- | --- | --- |
|  | *Baseline* | *Any new at follow-up* | *Total individuals* |  | *Baseline* | *Any new at follow-up* | *Total individuals* |
| Subcortical infarcts | 34 (2.7%) | 22 (1.8%) | 50 (4.0%) |  | 158 (12.6%) | 92 (7.3%) | 184 (14.7%) |
| Microbleeds | 167 (13.3%) | 160 (12.8%) | 273 (21.8%) |  | 266 (21.2%) | 312 (24.9%) | 364 (29.1%) |
| ePVS | 159 (12.7%) | 27 (2.2%) | 166 (13.3%) |  | 252 (20.1%) | 51 (4.1%) | 251 (20.0%) |

**Table S2: cSVD markers over time for the limited and severe baseline WMH burden groups.** Change in other cSVD markers besides WMH over the follow-up period for both the limited and severe baseline WMH burden group. Values refer to *n (%)* individuals with any amount of a marker at baseline, or any amount of a new marker at follow-up that was not present at baseline in the same location. Total individuals refers to the amount of *n* (%) individuals that either had a marker at baseline or a new marker at follow-up, but not at both timepoints, as to not count individuals twice. PVS: enlarged perivascular spaces.

**Sensitivity analyses of baseline DAWM and long-term WMH volume change**

In the results, both an increase in baseline DAWM sum rating and an increase in baseline WMH volume were associated with a larger increase in WMH volume at follow-up. Baseline WMH volume showed a stronger association than baseline DAWM in comparison. This raises the question what information DAWM can contribute while adjusting for baseline WMH. To this end, additional sensitivity analyses were conducted. Results of three sensitivity analyses are shown in Table S3. The primary model (Model A1) was additionally adjusted for baseline WMH in the limited WMH burden group, resulting in Model A2. As a result, a higher baseline DAWM sum rating was no longer significantly associated with a larger increase in WMH volume at follow-up (*B* (95% CI): 0.15 ml (-0.02, 0.33), *p*=.091).

Next, a subgroup with very low global WMH volumes at baseline was selected by taking the bottom quartile (25^th^ percentile) of the baseline WMH volumes normalized for total intracranial volume (<0.45%). At baseline, this subgroup of community-dwelling older adults had a low median [IQR] WMH volume of 4.48 ml [3.08, 5.50], yet had a relatively high mean (SD) DAWM sum rating of 1.59 ± 1.06. At follow-up, this subgroup showed a low median [IQR] WMH volume change of 0.99 ml [0.00, 2.31]. In this subgroup, a higher baseline DAWM sum rating adjusted for baseline WMH volume was associated with a larger increase in WMH volume at follow-up (*B*: 0.23 ml (0.02, 0.45), *p*=.034).

These outcomes of the sensitivity analyses suggest that baseline DAWM sum ratings explain variance above baseline WMH volumes in community-dwelling older adults with very low baseline WMH volumes.

|  | *Limited baseline WMH burden group (n=1253)* | |  | *Bottom quartile baseline WMH subgroup (n=627)* | |
| --- | --- | --- | --- | --- | --- |
|  | *Primary model (A1):*  *DAWM + standard covariates* | *Model A2:*  *DAWM + WMH + standard covariates* |  | *Model B1:*  *DAWM + standard covariates* | *Model B2:*  *DAWM + WMH + standard covariates* |
| *WMH vol. change (in ml) at FU*  *(B ± 95% CI)* | 0.26 (0.08, 45)* | 0.15 (-0.02, 0.33) |  | 0.28 (0.06, 0.49)* | 0.23 (0.02. 0.45)* |

**Table S3: Sensitivity analyses of associations between baseline DAWM and global WMH progression.** To test whether baseline DAWM sum ratings explain variance above baseline WMH volumes in community-dwelling older adults with limited and very low baseline WMH volumes, four sensitivity analyses were conducted. The table headers refer to which group is being analyzed, i.e. the limited burden group or the bottom quartile subgroup, and to the adjustments of each model. *B*-values (*±* 95% confidence intervals) indicate WMH volume changes (in ml) from baseline to follow-up per unit increase in baseline DAWM sum rating. Standard covariates: age, sex, total intracranial volume, hypertension, type two diabetes mellitus, smoking status, and BMI. *Vol.*: volume. *FU*: follow-up. **p*<.05.
